# Rare Oncogenic Fusions in Pediatric Central Nervous System Tumors: A Case Series and Literature Review

**DOI:** 10.3390/cancers16193344

**Published:** 2024-09-30

**Authors:** Melek Ahmed, Anne Sieben, Toon Van Genechten, Sasha Libbrecht, Nathalie Gilis, Mania De Praeter, Christophe Fricx, Pierluigi Calò, Claude Van Campenhout, Nicky D’Haene, Olivier De Witte, Léon C. Van Kempen, Martin Lammens, Isabelle Salmon, Laetitia Lebrun

**Affiliations:** 1 Division of Pathology, Antwerp University Hospital (UZA), 2650 Edegem, Belgium; 2 Instituut Born Bunge (IBB), 2610 Wilrijk, Belgium; 3 Division of Pediatric Oncology and Hematology, Antwerp University Hospital (UZA), 2650 Edegem, Belgium; 4 Center for Cell Therapy and Regenerative Medicine, Antwerp University Hospital (UZA), 2650 Edegem, Belgium; 5 Department of Neurosurgery, Université Libre de Bruxelles (ULB), Hôpital Universitaire de Bruxelles (HUB), CUB Hôpital Erasme, Erasme University Hospital, 1070 Brussels, Belgium; 6 Division of Neurosurgery, Antwerp University Hospital (UZA), 2650 Edegem, Belgium; 7 Department of Pediatrics, Université Libre de Bruxelles (ULB), Hôpital Universitaire de Bruxelles (HUB), CUB Hôpital Erasme, Erasme University Hospital, 1070 Brussels, Belgium; 8 Department of Pediatric Oncology and Hematology, Hôpital Universitaire des Enfants Reine Fabiola (HUDERF), 1020 Brussels, Belgium; 9 Department of Pathology, Université Libre de Bruxelles (ULB), Hôpital Universitaire de Bruxelles (HUB), CUB Hôpital Erasme, Erasme University Hospital, 1070 Brussels, Belgium; 10 DIAPath, Center for Microscopy and Molecular Imaging (CMMI), Université Libre de Bruxelles (ULB), 6041 Gosselies, Belgium; 11 Department of Pathology, Centre Universitaire Inter Regional d’Expertise en Anatomie Pathologique Hospitaliere (CurePath), 6040 Charleroi, Belgium

**Keywords:** oncogenic fusions, pediatric CNS tumors, DNA methylation, RNA sequencing, CNS WHO classification

## Abstract

**Simple Summary:**

Central Nervous System (CNS) pediatric tumors represent the most common solid tumors in children with a wide variability in terms of survival and therapeutic response. Unlike their adult counterparts, the mutational landscape of pediatric CNS tumors is mostly characterized by oncogenic fusions rather than multiple mutated genes. We report four pediatric cases associated with rare oncogenic fusions, providing an overview of oncogenic fusion pathogenesis, histological phenotype, diagnostic and theranostic impact. Our work underlines that most of these rare oncogenic fusions are not specific to a single morpho-molecular entity among the pediatric CNS tumors. Even within tumors harboring the same oncogenic fusions, a wide range of morphological, molecular and epigenetic entities can be observed. These findings highlight the need for caution when applying the fifth CNS WHO classification, as the vast majority of these fusions are not yet incorporated in the diagnosis, including grade evaluation and DNA methylation classification.

**Abstract:**

**Background and Objectives:** Central Nervous System (CNS) pediatric tumors represent the most common solid tumors in children with a wide variability in terms of survival and therapeutic response. By contrast to their adult counterpart, the mutational landscape of pediatric CNS tumors is characterized by oncogenic fusions rather than multiple mutated genes. CNS pediatric tumors associated with oncogenic fusions represent a complex landscape of tumors with wide radiological, morphological and clinical heterogeneity. In the fifth CNS WHO classification, there are few pediatric CNS tumors for which diagnosis is based on a single oncogenic fusion. This work aims to provide an overview of the impact of rare oncogenic fusions (*NTRK*, *ROS*, *ALK*, *MET*, *FGFR*, *RAF*, *MN1*, *BCOR* and *CIC* genes) on pathogenesis, histological phenotype, diagnostics and theranostics in pediatric CNS tumors. We report four cases of pediatric CNS tumors associated with *NTRK* (*n* = 2), *ROS* (*n* = 1) and *FGFR3* (*n* = 1) oncogenic fusion genes as a proof of concept. **Cases presentation and literature review:** The literature review and the cohort that we described here underline that most of these rare oncogenic fusions are not specific to a single morpho-molecular entity. Even within tumors harboring the same oncogenic fusions, a wide range of morphological, molecular and epigenetic entities can be observed. **Conclusions**: These findings highlight the need for caution when applying the fifth CNS WHO classification, as the vast majority of these fusions are not yet incorporated in the diagnosis, including grade evaluation and DNA methylation classification.

## 1. Introduction

Central Nervous System (CNS) pediatric tumors represent the most common solid tumors in children (incidence of 5.26/100,000), with a wide variability in terms of survival and therapeutic response [1,2]. The therapeutic strategy is mainly based on surgery, often achievable for lesions located in the cerebral hemispheres or posterior fossa, but is challenging for deep midline infiltrative tumors [3]. Chemo- and radiotherapy are classically used as adjuvant treatments or for progressive residual disease but are still associated with long-term morbidity and increased mortality [3]. Furthermore, there is a dramatic lack of molecular targeted treatments [2].

In 2021, the fifth World Health Organization Classification of Tumors of the Central Nervous System (WHO CNS5) introduced specific pediatric CNS tumor entities such as “pediatric-type diffuse gliomas”, underlying the specificity of these tumors. This specific histo-molecular classification improved the stratification of these tumors, underlining their complexity and heterogeneity.

By contrast to their adult counterparts, the mutational landscape of pediatric tumors is characterized by oncogenic fusions rather than multiple mutated genes. Gene fusions can result in a hybrid protein that is constitutively active or has altered function. The most common example is the *KIAA1549: BRAF* fusion, first described in 2010 by Jones et al., which results from a tandem duplication of the *BRAF* gene and represents the molecular hallmark of pilocytic astrocytoma. Since then, the omics revolution has enabled us to find many other driver gene transcript fusions with various fusion partners and genomic breakpoints, including, but not limited to, *(B)RAF*, *ROS1*, *NTRK1/2/3*, *FGFR 1/2/3*, *EGFR* and *PDGFRA* [4,5].

Pediatric CNS tumors associated with oncogenic fusions represent a complex landscape of tumors with wide radiological, morphological and clinical heterogeneity. In the WHO CNS5, there are few pediatric CNS tumors for which diagnosis is based on a single oncogenic fusion. In 2021, the new entity named “supratentorial ependymoma *YAP1*-fusion positive” was introduced by the WHO, defining a group of tumors in infants with a specific morphology and a usually good prognosis [6]. Emerging evidence in the literature and DNA methylation classifications suggests that other histo-molecular entities could also be defined by a single-driver oncogenic fusion [7,8]. However, data in the literature are lacking regarding the real impact on diagnosis since some fusions are shared by several low-grade (LG) and high-grade (HG) pediatric CNS tumors. Therefore, challenges remain regarding the diagnostic, prognostic and theranostic impact of these fusions.

This work aims to provide an overview of the impact of rare oncogenic fusions on pathogenesis, histological phenotype, diagnostics and theranostics in pediatric CNS tumors. Four pediatric CNS tumor cases associated with rare oncogenic fusions are used as proof of concept of the complexity these oncogenic fusions provide in the practical routine management of these patients.

## 2. Cases Description

- *NTRK*-fused cases

**Figure 1:**
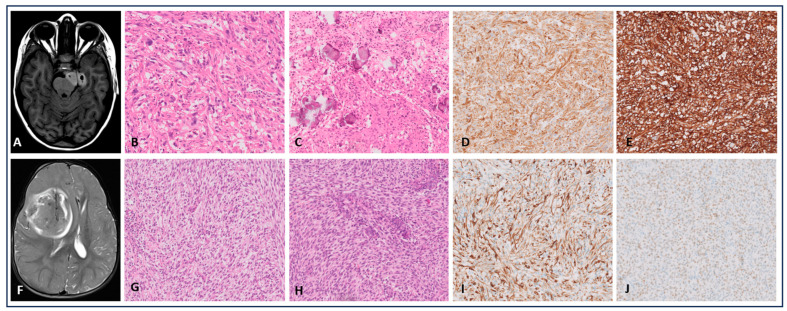
*NTRK*-fused cases (**A**–**J**); MRI and 20× magnified histological images. Case 1 (**A**–**E**): (**A**) Axial T1-weighted MRI showed a lesion in the left mesial temporal lobe, (**B**,**C**). HE: A pleomorphic tumor with spindle-shaped and multinucleated giant cells, and calcifications were observed. (**D**) The tumor was GFAP-positive. (**E**) Diffuse cytoplasmic and membranous positivity for CD34. Case 2 (**F**–**J**): (**F**) Axial T2-weighted MRI showed a large tumoral mass in the right frontal lobe. (**G**,**H**) The tumor was composed of spindle-shaped and focally gemistocytic cells with high mitotic activity; no necrosis was observed. (**I**,**J**) GFAP and olig2 were focally positive.

The first case concerns a 6.5-year-old girl who consulted for focal seizures with impaired consciousness. Magnetic Resonance Imaging (MRI) showed an intra-axial left mesial temporal tumor with solid and several small cystic components. The lesion was conspicuously hyperintense on T1-weighted images, and showed only modest contrast enhancement (Figure 1). Subtotal surgery was performed. Histopathological examination revealed a well-defined tumor. The lesion was heterogeneous with a predominant storiform growth pattern. The tumor cells were spindle-shaped with intermixed giant cells. Throughout the specimen, lymphocytes, plasma cells and eosinophilic granular bodies were present. There were calcifications, but no necrosis was observed. The mitotic activity was low (1 mitotic figure/2.3 mm^2^). Most of the tumor cells were positive for the glial fibrillary acidic protein (GFAP), CD34 (diffuse cytoplasmic and membranous positivity), with focal expression of Neu-N and synaptophysin on immunohistochemical analysis (IHC). Based on the morphological aspect and the IHC profile, we proposed the diagnosis of a pleomorphic xanthoastrocytoma (PXA), WHO grade 2. Additional tyrosine kinase (panTRK) IHC showed strong cytoplasmic staining with membranous accentuation. Targeted next-generation sequencing (NGS) (Supplementary Material S1) showed no *BRAF*, *RAS* or other mutations. RNA sequencing (Supplementary Material S1) revealed a *TPM4* (*exon 7*)::*NTRK2* (*exon 15*) fusion. Genome-wide DNA methylation analysis was performed using the Illumina Infinium MethylationEPIC BeadChips, but unfortunately, DNA methylation testing was not contributive. The patient received no adjuvant therapy [9]. To date, five years after surgical treatment, no progression has been seen on MRI.

The second case depicts a 1-year-old girl who presented with altered consciousness after a fall. MRI showed a large tumoral mass in the right frontal lobe with associated hemorrhage. Total resection was performed. Histopathological examination showed variably cellular sheets and bundles of plump spindled tumor cells with eosinophilic cytoplasm, monomorphic oval nuclei with a prominent nucleolus and focal pseudorosettes. There was no necrosis present. Focally, these cells were larger, with a gemistocytic appearance, eosinophilic cytoplasm and an eccentric nucleus. Extensive microvascular proliferation with increased amounts of lymphocytes in the perivascular spaces was noted (Figure 1). The mitotic activity was high, with nine mitoses per 2.3 mm^2^ and a Ki-67 proliferative index nearing 10% in the hotspots. The tumor cells were positive for vimentin and S100, and focally positive for GFAP and olig2. INI1 and BAP1 showed retained nuclear staining. Regarding molecular analyses, targeted NGS showed no gene mutations, while RNA sequencing analysis (Supplementary Material S1) showed an *ETV6 (exon 5)::NTRK3* (*exon 15*) fusion. Methylation profiling (version 11b4 of the Heidelberg classifier by DKFZ) showed a match with methylation class ‘Infantile hemispheric glioma’, with a calibrated score (cs) of 0.92.

Based on the WHO CNS5 edition this tumor was classified as a pediatric-type diffuse high-grade glioma, subtype ‘Infant-type Hemispheric Glioma (IHG)’.

No adjuvant treatment was given. To date, four years after surgery MRI follow-up has shown no disease recurrence.

- *ROS1*-fused case

An 8-year-old boy presented with a history of tremor, balance disorders, mild headache, weight loss and fatigue for several months. MRI showed two T2-hyperintense lesions in the pineal region and in the wall of the lateral ventricle. Debulking was performed, leaving a large unresectable residual tumor behind.

**Figure 2:**
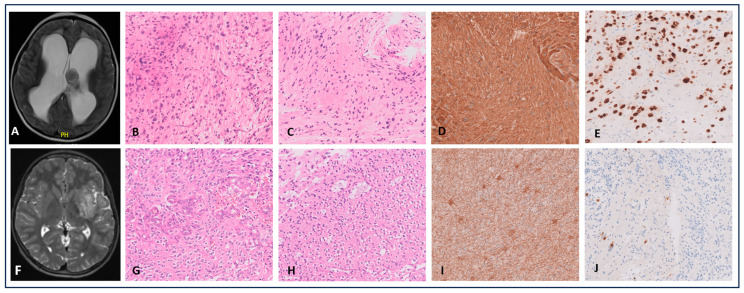
*ROS1*-fused (**A**–**E**) and *FGFR3*-fused (**F**–**J**) cases; MRI and 20× magnified histological images. Case 3: (**A**–**E**): Axial T2-weighted MRI showed two hyperintense lesions in the pineal region and in the wall of the lateral ventricle. (**B**,**C**): HE showed a biphasic tumor with piloid features, eosinophilic granular bodies and calcifications. (**D**,**E**): GFAP and olig2 were at least partially positive. Case 4 (**F**–**J**): (**F**). Axial T2-weighted MRI showed a hyperintense tumoral mass in the left temporal lobe. (**G**,**H**): HE showed microvascular proliferation; no necrosis was seen. The tumor cells were ‘oligodendroglial-like’ and neurons were observed in mucoïd pools. (**I**): GFAP showed sporadically staining cells. (**J**): Some rare NeuN-positive cells were seen.

Microscopic examination showed low to moderate cellularity. The tumor was biphasic, with alternating areas of bipolar cells and loose to microcystic regions. Focally, some pigment deposition, eosinophilic granular bodies and small calcifications were seen. The tumor cells were rather piloid, with round to elongated nuclei. There was no neuronal component. No mitotic activity nor necrosis was observed (Figure 2A–E). The tumor cells were positive for GFAP and partially positive for olig2. ATRX staining was preserved. Ki-67 showed a low proliferative index estimated at 1%. RNA sequencing analysis was performed and a *GOPC* (exon8)*::ROS1* (exon 35) fusion was found. No additional molecular alterations were found with targeted NGS (Supplementary Material S1).

Genome-wide methylation analysis (version 12.5 of the Heidelberg classifier by DKFZ) showed a calibrated score of 0.64 for pilocytic astrocytoma. Based on morphology, immunohistochemistry and molecular and epigenetic data the diagnosis of a pilocytic astrocytoma (CNS WHO grade 1) with an *ROS1* fusion was established.

The patient received adjuvant vincristine and carboplatinum (according to the SIOP LGG 2004 protocol) [10]. The most recent MRI (9 months after the initial diagnosis) showed residual tumor without progression.

Interestingly, reanalysis with the 12.8 version of the DKFZ classifier showed a calibrated score of 0.45 for pilocytic astrocytoma.

- *FGFR3*-fused case

A 12-year-old boy presented to the emergency department with progressive headache, absence seizures and strange taste perception. MRI showed a tumoral mass in the left temporal lobe with multiple T2-hyperintense cystic components and a solid contrast-enhancing component. The patient underwent subtotal resection. Microscopic examination showed a polymorphous tumor. Both well-circumscribed and infiltrative growth patterns associated with trabecular and nested architecture were observed. The tumoral cells in the trabecular regions harbored enlarged, irregular, hyperchromatic nuclei while in the diffuse growing areas they were rather “oligodendrocyte-like”, showing small, uniform nuclei and a perinuclear halo. Areas with prominent microvascular proliferation, as well as areas with fine vascular networks, were present. There was increased mitotic activity (estimated to be 1 mitosis/2.3 mm^2^) in the trabecular areas. Calcifications were observed but no necrosis was seen (Figure 2F–J). Olig2 and synaptophysin IHC were positive in the tumor cells while GFAP was mostly negative. CD34 showed no extravascular positivity and NeuN showed a few entrapped neurons. Ki-67 showed a proliferation rate of 3–5%. No mutations were detected by targeted NGS (Supplementary Material S1). RNA sequencing (Supplementary Material S1) analysis showed an *FGFR3::TACC3* fusion. Finally, genome-wide DNA methylation profiling was performed. Using the Heidelberg DNA-methylation classifier (v12.5), the case was classified as “Low Grade Glioneuronal tumor” (with a csof 0.86). Further subtyping was not possible based on the methylation profile as the scores were <0.5. Integration of the morphologic, immunohistochemical, genetic and epigenetic data led to the diagnosis of “Glioneuronal tumor, not elsewhere classified (NEC) with a *FGFR3::TACC3* fusion, favor WHO grade 2”. No adjuvant therapy was given. Nearly one year after the initial presentation, follow-up MRI showed no progression.

Recently, the case was reanalyzed using v12.8 of the DKFZ brain classifier. This led to further subtyping of this lesion as a dysembryoplastic neuroepithelial tumor methylation class (MC) (with cs of 0.70).

## 3. Literature Review

### 3.1. The Landscape of Kinase Fusions [11]

- *ALK* fusions

The *ALK* (anaplastic lymphoma kinase) gene (located at 2p23.2-p23.1) encodes a receptor tyrosine kinase (RTK) which plays a role in the regulation of the Wnt/beta-catenin pathways. Under physiological conditions, ALK activates pathways such as MAPK, PI3K/AKT/mTOR and JAK/STAT in nerve cells [12].

The *ALK* gene can be rearranged, amplified or mutated in pediatric CNS tumors [13]. *ALK* fusions with *PPP1CB*, *CCDC88A*, *EML4*, *HIP1L*, *PRKAR2A*, *SPTBN1*, *MAD1L1*, *MAP2*, *MSI2*, *SPECC1L1*, *SYNDIG1L*, *ZC3H7A* and *CLIP2A* in which a functional *ALK* kinase domain is preserved are the most commonly described in IHGs [11]. The fusion partners have in common that they can mediate ligand-independent dimerization and subsequent activation of the ALK kinase domain.

According to the WHO CNS5 classification [14], the presence of an *ALK*, or other receptor tyrosine kinase (RTK), oncogenic fusion is one of the essential diagnostic criteria for the diagnosis of IHG. Other essential diagnostic criteria include cellular astrocytoma, early childhood (first year of life) and cerebral hemispheric location. However, *ALK* gene fusions can also be detected in LGGs. In contrast, *ROS1*/*NTRK*/*MET* fusions appear to be more likely associated with HGGs according to a large international cohort published in 2019 [15]. In the study of Guerrero-Stucklin et al. [15], infant gliomas with different *ALK* gene fusions were reported. Interestingly, infants with *ALK*-fused, morphologically HG gliomas have a worse outcome (42.9% overall survival in *ALK*-fused HG gliomas at a median follow-up of 3 years versus overall survival of 100% in *ALK*-fused LG gliomas at a median follow-up of 5 years) and tend to be diagnosed at an older age (median = 5.0 months versus 1.6 months old) compared to *ALK*-fused LG gliomas [13,15].

Lorlatinib, a third-generation *ALK* inhibitor, has shown a treatment advantage over chemotherapy in *ALK*-rearranged gliomas in mouse models. Other *ALK* inhibitors approved by the United States Food and Drug Administration (FDA) and European Medicines Agency (EMA) include crizotinib, ceritinib, alectinib and brigatinib [13]. Among these, alectinib has been shown to have the best CNS penetration [16,17,18,19,20]. The above-mentioned *ALK*-inhibitors are approved both by FDA and EMA for non-small cell lung carcinoma (NSCLC). To our best knowledge there are no clinical trials registered to explore the role of *ALK*-inhibitors in the specific setting of pediatric glioma. Nevertheless, basket precision medicine trials are aiming to treat patients according to the molecular profile of the tumor. In this setting, *ALK*-inhibitors can be administered in medical need programs [21,22].

- *ROS1* fusions

The *ROS1* gene (located at 6p22.1) encodes a proto-oncogene 1 transmembrane tyrosine protein kinase that binds to growth factors and undergoes dimerization and phosphorylation with transmission of growth signals downstream. Rearrangement of this gene leads to a constitutively active ligand-independent tyrosine kinase fusion product that has been proven to be sufficient for tumorigenesis [23]. Both ALK and ROS1 receptors activate the MAPK, as well as the JAK/STAT and PI3K/AKT/mTOR pathways [2].

Six different *ROS1* fusion partners have already been described in pediatric CNS tumors (+/−7% of pediatric CNS tumors): *GOPC*, *ARCN1*, *CHCHD3*, *ZCCHC8*, *TPR* and *CEP85L* [2,23,24,25]. The *GOPC* gene is the most frequent fusion gene partner [26]; it is a ubiquitously expressed gene that encodes for a dimeric protein associated with the Golgi apparatus. The resulting protein of the *GOPC-ROS1* fusion gene has been proven to be sufficient to initiate neoplastic transformation [2,20]. Although the breakpoints are different, all the *ROS1* fusions retain the kinase domain. The resulting fusion protein results in cytoplasmic activity of the ROS1 kinase domain [2].

DNA methylation analyses of a large case series revealed that *ROS1*-fused tumors clustered into different glioma groups, suggesting that *ROS1* fusions are not specific to a single glioma type. Interestingly, most of the patients with *ROS1* gene fusions were children, and mostly infants, with a high frequency of *ROS1* gene fusions present in the IHG methylation class [25]. However, *ROS1* gene fusions can also appear in cases that are morphologically and (epi)genetically pilocytic astrocytoma or *IDH*-wildtype glioblastoma. *ROS1* fusions have been further described in meningioma, ependymoma, LGG and glioneuronal tumors [23,26]. The heterogeneity of morphological findings underlines the fact that *ROS1* gene fusions are not pathognomonic for IHG, nor limited to the pediatric population [25]. Since they are not pathognomonic, they have limited diagnostic value on their own. We have therefore to be careful with the criteria used in the fifth CNS WHO classification to diagnose IHG, which include the following: cellular astrocytoma, hemispheric location and early childhood associated with *ROS1*, *ALK*, *NTRK* or *MET* fusions. These criteria can lead to overdiagnosis of HGG. Interestingly, the type of RTK fusion seems to have a prognostic implication given that *ALK*-fused IHGs have a better outcome than *ROS1*-fused IHGs [15].

Similarly to *ALK*, *ROS1* fusion proteins are potentially targetable [25]. Different ROS1 inhibitors can potentially play a therapeutic role in *ROS1*-fused glioma. Crizotinib and entrectinib are tyrosine kinase inhibitors (TKI) that target *ALK*, *ROS1* and *MET* receptor tyrosine kinase. Lorlatinib is a more recently developed tyrosine kinase inhibitor with better blood–brain barrier permeability which has already shown better outcomes in lung cancer patients with brain metastases [23]. As mentioned previously, these drugs are FDA- and EMA-approved for NSCLC treatment, but there are just a few case reports mentioning good response to entrectinib in *ROS1*-fused glioma [26].

- *NTRK* fusions

The tropomyosin receptor kinase (TRK) family is composed of TRKA, TRKB and TRKC, which are a group of cell membrane receptors encoded by neurotrophic TRK (NTRK) genes *NTRK1*, *NTRK2* and *NTRK3*, highly expressed in neural tissue [2,27,28]. The *NTRK1*, *NTRK2* and *NTRK3* genes are located on chromosomes 1 (1p22), 9 (9p22) and 15 (15q25), respectively [29]. The TRK receptors and their downstream pathways are involved in neuronal development, synaptic plasticity, cell survival, growth, differentiation and proliferation [2,5,27,29,30]. TRK can become oncogenic in different ways, most commonly as a result of structural chromosomal rearrangements leading to gene fusions, in addition to splice variants and mutations [2,28,29]. Multiple fusion partners for the *NTRK* gene have been identified [28,29]. *NTRK* gene fusions occur by either intra- or inter-chromosomal rearrangement [28,30]. Oncogenic *NTRK* gene fusions result in aberrant ligand-independent TRK receptor dimerization and constitutive activation of TRK signaling pathways. This leads to upregulated proliferation and resistance to apoptosis. Multiple in vivo models support the hypothesis that *NTRK* gene fusions can drive gliomagenesis/tumorigenesis [28].

Among pediatric CNS tumors, the incidence of *NTRK* gene fusions is quite variable between different tumor types: 5.3% of pHGGs, 4% of diffuse intrinsic pontine glioma (DIPG) and 40% of non-brainstem HGG in infants (younger than 3 years old) show an *NTRK* fusion [28]. This fusion is not specific to HGGs since it can also occur in pilocytic astrocytoma [2]. Indeed, the morphological landscape and grade of *NTRK*-fused gliomas are highly variable. Different histological entities, both LG and HG, can harbor an *NTRK* fusion. Previous studies have described this gene fusion transcript in glioblastoma, high-grade glioma, high-grade glioma with features of pleomorphic xanthoastrocytoma (PXA), anaplastic astrocytoma, anaplastic ependymoma, glioma with anaplastic features, (anaplastic) pilocytic astrocytoma, ganglioglioma, diffuse astrocytoma, desmoplastic infantile ganglioglioma and LGG [5,28,29]. In the infantile population, this fusion is mostly an isolated molecular feature [29], but in some pediatric cases it co-occurs with other molecular alterations [28]. The fifth CNS WHO classification mentions *NTRK* fusions as a molecular feature of IHG, diffuse low-grade glioma, MAPK pathway-altered and PXA [14]. From an epigenetic point of view, most *NTRK*-fused gliomas to date have not shown a confident match with specific MCs [28,30].

Clinically, *NTRK* fusions are interesting due to FDA-approved *TRK*-inhibitors, such as larotrectinib and entrectinib [27,28], followed by crizotinib and cabozantinib, which are multikinase inhibitors [27]. Studies have already demonstrated successful responses to entrectinib for *ETV6-NTRK3*-fused IHGs [31]. Further studies have to investigate the possible acquired resistance to these drugs.

- *MET* fusions

The proto-oncogene mesenchymal–epithelial transition factor (MET) encodes for an RTK, which activates the MAPK, PI3K/AKT/mTOR, SRC and JAK/STAT pathways associated with the activation of cell proliferation, invasion and angiogenesis [32]. *MET* fusions are the least frequently occurring RTK gene fusions with three oncogenic fusions described in pediatric CNS tumors: *CLIP2-MET*, *TFG-MET* and *PTPRZ1-MET* [2].

Similar to *ALK*, *ROS1* and *NTRK*, *MET* gene fusions are integrated in the essential diagnostic criteria of the IHGs in the fifth CNS WHO classification. In a cohort of 53 pediatric glioblastomas, 10% harbored MET oncogenic fusions [33]. However, *MET* fusions are not specific to pediatric-type diffuse HGG [34], and have been described in pediatric LGG such as pediatric-type glioneuronal tumors (proposed to be named ‘glioneuronal tumor kinase-fused’ (GNT_KinF_A)) [35].

MET inhibitors have already shown promise in the treatment of *PTPRZ1-MET* fusion-driven pediatric glioblastoma, with improved clinical and radiological responses over a period of 2 months [33]. Recently, Zuckermann et al. suggested a better efficacy of capmatinib associated with radiotherapy than crizotinib and radiotherapy or cabozantinib and radiotherapy in orthotopic mouse models harboring distinct MET fusion-associated IHG [36].

- *FGFR* fusions

The fibroblast growth factor receptor (*FGFR*) gene family consists of four transmembrane tyrosine kinase receptors (FGFR1-4) and represents an important RTK signaling pathway. FGFR dimerizes in the presence of its ligands and triggers downstream signaling pathways. These pathways include the MAPK and PI3K/AKT/mTOR pathways. FGFR signaling plays a fundamental role in CNS embryonal development; angiogenesis; and tumor cell migration, differentiation, proliferation and survival [37].

*FGFR* gene fusions mostly involve one of the three TACC (Transforming Acidic Coiled-Coil Containing Protein) genes that encodes the centrosomal proteins TACC1, TACC2 or TACC3. These fusions give rise to constitutive FGFR activity and downstream MAPK/PI3K/mTOR pathway activation. *FGFR3-TACC3* is relatively frequently seen in glioblastoma, *IDH*-wildtype, with a frequency of around 3 to 4% [37].

The fifth CNS WHO classification mentions *FGFR* fusions as an essential or desirable feature in extraventricular neurocytoma (*FGFR1-TACC1*), dysembryoplastic neuroepithelial tumor (DNET, *FGFR1* fusion or other *FGFR1* alterations), polymorphous low-grade neuroepithelial tumor of the young (PLNTY, *FGFR2-CTNNA3* and *FGFR3* fusions) and multinodular and vacuolating neuronal tumor (MVNT, rarely *FGFR2* fusions). Diffuse LGG, MAPK-pathway-altered, is also a possible differential diagnosis of *FGFR3*-fused tumors [14].

In the pediatric population, *FGFR3* fusions occur mostly, but not exclusively, in LGG, while in the adult population they are mostly associated with HGG. *FGFR3-TACC3* is by far the most prevalent fusion. Both LGG and HGG, *FGFR3*-fused, exhibit typical histological features: oligodendrocyte-like cells, a ‘chicken-wire’ capillary network and microcalcifications [37,38,39,40]. Métais et al. also reported that these features are shared by some other pediatric-type diffuse LGG (i.e., PLNTY and a subgroup of diffuse LGG, MAPK pathway-altered) [40].

Patients younger than 40 years of age diagnosed with an *FGFR3-TACC3* glioma showed a significantly better progression-free survival (PFS) and overall survival (OS) compared to non-*FGFR3-TACC3*-fused gliomas. Patients with morphological HG *FGFR3*-fused tumors had a worse PFS but a comparable OS to those with LG morphology. Furthermore, cases with concomitant p*TERT* mutation had a worse prognosis [40].

Since diffuse gliomas with *FGFR3-TACC3* fusion are characterized by their epigenetic and clinical heterogeneity, they are not recognized as a distinct entity by the fifth CNS WHO classification. The presence of an *FGFR3-TACC3* fusion in a young patient does not lead to the diagnosis of a specific tumor type and/or grade. Methylation profiling proposes alternative diagnoses such as DNET and GG. One should be cautious when establishing the latter diagnoses based on methylation analysis alone, since the WHO criteria for diagnosis still include ‘essential’ histomorphological features [40].

*FGFR2* gene fusions are seen in a recently described entity, the PLNTY. Infiltrative growth pattern, oligodendrocyte-like morphology and frequent calcifications are common morphological features of this tumor [14]. Regional expression of CD34 by tumor cells and by ramified neural cells in the associated cerebral cortex is an essential criterion for this entity. These tumors also harbor a *BRAF* p.V600E mutation, *FGFR2* or *FGFR3* fusions or other MAPK pathway-driving genetic alterations [14,37].

Regarding the *FGFR1* gene, *FGFR1-TACC1* is most commonly associated with LG glioma, mostly extraventricular neurocytoma (EVN), but other *FGFR* fusions can also be seen in EVN [41].

The oral multikinase inhibitor regorafenib already showed promising results in recurrent glioblastoma in adults with *FGFR3-TACC3* fusion [42]. In other tumor types, erdafitinib, rogaratinib, infigratinib, dovitinib and the monoclonal antibody vofatamab are being tested in preclinical models and clinical trials [5,39]. Further research is needed to explore the therapeutic efficacy of *FGFR* inhibitors in young patients with glioma.

- *RAF* fusions

The RAF (rapidly accelerated fibrosarcoma) proto-oncogene serine/threonine kinase family encompasses fusion proteins which are described to have various and different fusion partners [2].

In pediatric LGGs, the downstream MAPK pathway activation is mainly caused by *KIAA1549-BRAF* fusion (35% of LGG) and *BRAF* p.(V600E) mutation (15% of LGG). Other activating events are rather rare and include other *BRAF* (non-*KIAA1549)* fusions and *CRAF* (*RAF1*) fusions (<2% of LGG), in addition to the previously described alterations in *ALK*, *ROS1*, *NTRK*, *MET* and *FGFR* [3,43].

To date, more than 10 different BRAF fusion partners, other than *KIAA1549*, have been described in pediatric CNS tumors, including *FXR1*, *MACF1*, *FAM131B* [43,44], *NFR1*, *TMEM106B*, *RNF130*, *CLCN6*, *MKRN1*, *CTTNBP2* and *GNA11* [44,45]. These rare *BRAF* fusions have been reported in grade 2 and 3 pleomorphic xanthoastrocytoma, pilocytic astrocytoma and low-grade glial/glioneuronal tumors, not otherwise specified (NOS) [43,45], and constitute a small minority of all BRAF-fused gliomas.

*RAF1* gene fusions have been shown to also activate the MAPK/ERK pathway, in addition to the PI3K/AKT/mTOR pathway [45,46]. Their clinical implication remains unclear, mainly due to the limited occurrence of these fusions [46]. Several RAF1 fusion partners have already been described: *QKI*, *FYCO1*, *TRIM33*, *SRGAP3*, *NF1A*, *ATG7* and *TRAK1* [3,44,45,46,47]. To date, the cases are limited to low-grade glial/glioneuronal tumors [43,44,45,46,47]. The histological tumor types encompass pilocytic astrocytoma [44,46,47]; pleomorphic xanthoastrocytoma [45]; low-grade glial-glioneuronal tumors, not otherwise specified (NOS); desmoplastic infantile ganglioglioma (DIG); and diffuse leptomeningeal glioneuronal tumor (DLGNT) [46].

The limited experience with *RAF1* fusions due to their rarity underscores the need for future long-term follow-up to determine the diagnostic and prognostic implications of these fusions [46].

Unlike *BRAF* fusions, first- and second-generation *RAF* inhibitors are not effective in *RAF1*-fused glioma [2,46]. However, in vitro assays have identified at least partial responses to selumetinib and trametinib (*MEK*-inhibitors), and sorafenib (a multikinase inhibitor) [44]. To date, no clinical studies are available investigating the therapeutic relevance of *RAF1* fusion.

### 3.2. The Landscape of Transcription Regulators

- *MN1* fusions

The Meningioma 1 (*MN1*) proto-oncogene (located at 22q12.1) is a transcriptional coregulator that has been shown to overstabilize the tSWI/SNF chromatin remodeling complexes when overexpressed [48], and was first described in a balanced translocation (4;22) in meningioma. *MN1* alterations have also been described in acute myeloid leukemia (AML) with a potential prognostic impact [49,50].

*MN1* is implicated in the maturation and normal function of calvarial osteoblasts associated with the normal development of the membranous bones of the skull. MN1 could also have a tumor suppressor gene function by interacting with the repression of cell proliferation [51]. However, the exact oncogenic role is unknown [2].

In the fifth CNS WHO classification, MN1 is part of the diagnosis of a “circumscribed astrocytoma”, Astroblastoma *MN1*-altered, characterized by fusions of *MN1* with *BEND2* or *CXXC5* genes. BEN domains in BEND2 are implicated in DNA remodeling and neural transcriptional regulation while CXXC5, a member of the zinc-finger CXXC family, is a transcriptional activator involved in myelopoiesis and oligodendrocyte differentiation [2,14,52].

Interestingly, *MN1-BEND2* and *MN1-CXXC5* seem to have different clinical, morphological and epigenetic characterization, suggesting that the fusion partner impacts the phenotype. More than 60% of MN1 fusion-positive CNS tumors show astroblastoma morphology characterized by astroblastic perivascular pseudorosettes but astroblastomas, such *MN1*-altered astroblastomas, remain a rare, heterogenous and poorly characterized tumor group. *MN1-CXXC5*-fused CNS tumors more frequently harbor a «PNET»-like morphology [53].

The two main MN1 alterations (*MN1:BEND2* and *MN1:CXXC5*) are adjacent but cluster differently in DNA methylation profile analysis [54,55,56]. The new version of the DNA methylation classifier (v12.8) includes two different MCs: the MC astroblastoma, MN1-altered and MN1-BEND2-fused, and the MC neuroepithelial tumor, *MN1-CXXC5*-fused (https://www.molecularneuropathology.org/mnp/classifiers, accessed on 15 March 2023).

From a clinical point of view, with a favorable overall survival (5-year survival up to 90%), the median PFS seems different depending on the molecular rearrangement (60% *CXXC5* > 40% *BEND2*-fused) [53,57].

- *PATZ1* fusions

The POZ/BTB and AT-Hook-Containing Zinc Finger Protein (*PATZ1*) gene is localized on chromosome 22q12. It has been described as part of a network of transcription factors that maintain the ‘stemness’ of embryonic stem cells by inhibiting neural differentiation, and a regulator of cellular reprogramming [58,59]. It could act both as an activator or repressor of transcription, depending on the cellular context [58]. It plays an important role in development, cell proliferation, senescence and apoptosis [60].

Recently, a molecularly distinct group of predominantly pediatric CNS neoplasms with *PATZ1* fusions was described. This group of tumors revealed mainly *MN1-PATZ1* or *EWSR1-PATZ1* fusions [2,5,52,58]. Morphologically, these cases are quite heterogeneous and polyphenotypic. The HG astrocytoma morphology was the most commonly described. Ganglioglioma, ependymoma-, subependymoma-like, LG glial and glioneuronal morphology were also reported. A recent report of seven cases described a spindle cell sarcoma morphology. Beside a heterogeneous morphology, the immunophenotypic features were also broad [5,58,61].

Further analysis of these tumor groups using t-distributed stochastic neighbor embedding (t-SNE) showed distinct grouping of this set of tumors. There was no overlap with the recently described high-grade neuroepithelial tumors with *MN1* fusions, which are characterized by *MN1-BEND2* and *MN1-CXXC5*. The tumors score poorly (<0.6 calibrated score) for the currently known entities. This tumor group was provisionally called ‘neuroepithelial tumor with *PATZ1* fusion’ [58]. Interestingly, unsupervised clustering analysis of gene expression profiles did not show homogeneity of gene expression with two distinct transcriptional subgroups correlating with the morphological aspect (sarcomatous vs. others) [61].

The copy number variation plot of these tumors was rather ‘quiet’ with the notable presence of recurrent structural copy number variations in chromosome 22, which was seen in 98% of the described cases [58]. They thus defined these *PATZ1*-fused neuroepithelial tumors as defined by y chromosome 22 chromothripsis.

From a clinical point of view, the *PATZ1*-fused tumors were mostly supratentorial and the majority of the tumors occurred in patients under 18 years of age. Patient outcome suggested an intermediate malignancy grade [58,61].

Given the fact that to date only a limited number of cases have been described, further studies are needed to further understand the biology, cellular origin, therapeutic consequences and clinical outcome of these tumors. The targetability of this fusion has to date only been tested in the context of drug screening on cell lines [58].

- *BCOR* fusions

The BCL6 corepressor (BCOR) is a POZ/zinc-finger transcription repressor required for germinal center formation and is associated with embryonic development [2,62]. Moreover, the BCOR protein interacts with the class I and II histone deacetylases (HDACs) [63].

*BCOR* internal tandem duplication (ITD) has already been included in the WHO CNS5 for the diagnosis of “CNS tumor with BCOR ITD”. This embryonal tumor is almost exclusively found in young children [64].

*BCOR* fusions have also been identified in pediatric CNS tumors with two different gene fusion partners: *EP300* and *CREBBP*. These genes are both histone acetyltransferases associated with the regulation of the gene’s transcription [2].

Few cases of *EP300*-*BCOR*-fused CNS tumors have been described in the literature. A recent study compared the 23 reported cases to date and concluded that these tumors are not exclusively found in children (median age 30 years old) [64]. The morphological features are similar to the CNS tumor with *BCOR* ITD such as the microcystic aspect with myxoïd changes, pseudo-ependymal features and the oligodendrocyte-like pattern.

Interestingly, the DNA methylation classification provides a specific MC for BCOR/BCORL1-fused CNS tumors, distinct from the BCOR-ITD CNS tumors. However, it has to be determined if both MCs represent distinct histo-molecular entities.

Finally, no sufficient data are available to make robust conclusions regarding the prognostic impact of the *EP300*-*BCOR* fusion. *BCOR* ITD-CNS tumors seem to have a higher rate of recurrence than *EP300*-*BCOR*-fused CNS tumors (65% vs. 53%).

Regarding the *BCOR*–*CREBBP* fusion, data are very limited since only a few cases have been reported. There is a morphological overlap between *BCOR–CREBBP*- and *BCOR* ITD/*EP300*-*BCOR*-fused CNS tumors including oligodendrocyte- or ependymoma-like morphology, microcystic changes and also focal calcifications associated with anaplastic features [65].

- *CIC* fusions

The capicua transcriptional repressor (*CIC*) gene (located on chromosome 19q13.2) encodes for a transcription factor (transcription repressor) [66]. With the DNA-binding high-mobility group (HMG) box domain, *CIC* inhibits ETV1/4/5 expression and counteracts the activation of genes downstream of RTK signaling [67]. Loss of function and rearrangements of the *CIC* gene lead to enhancement of the transcriptional activity of CIC downstream targets [67].

*CIC* gene fusions with *NUTM1* have been described by Sturm et al. in “CNS Ewing sarcoma family tumor with *CIC* alteration” and represent the vast majority of CNS *CIC*-rearranged sarcomas [8,66]. These tumors are characterized by their high-grade aspects and variable morphology with small cell phenotype, with alveolar and fascicular patterns of growth being typical [66].

The WHO CNS5 introduced this new entity of “*CIC*-rearranged sarcoma” among the CNS mesenchymal tumors. Various partners are described such as *DUX4*, but also *FOXO4*, *LEUTX*, *NUTM1* and *NUTM2A*.

Other fusion partners have been described since but are limited to very few cases reported. *CIC-LEUTX* was already reported in one CNS embryonal tumor and in an anaplastic pleomorphic astrocytoma [2,8]. Sievers et al. described nine cases of *CIC-LEUTX*-fused pediatric CNS tumors that harbored heterogeneous morphological aspects but shared common DNA methylation signatures [67]. All these cases arose in early childhood and were associated with early recurrences [67]. However, it has to be confirmed if the “high-grade neuroepithelial tumor *CIC* fusion-positive” represents a specific entity, different from the “*CIC*-rearranged sarcomas”.

## 4. Discussion

Research by several groups has demonstrated the fundamental role of molecular analysis in the diagnostic work-up and therapeutic decision-making for pediatric CNS tumors [68]. Genetic and epigenetic information can help in different ways. The molecular signature can be used for confirmation of a certain diagnosis or for further refinement of a diagnostic group. Furthermore, it can lead to the consideration of a different diagnostic entity. The question remains open whether fusion-driven pediatric CNS neoplasms should be diagnosed based on their histopathological features, genetic alterations or epigenetic profiling [68]. Hereby, we would like to emphasize the importance of an integrated diagnosis, where both the histological classification and molecular findings are reported, as recommended by the fifth edition of the WHO classification of CNS tumors [3,69].

To note, some additional fusions of diagnostic importance exist (e.g., *KIAA1549-BRAF*, *MYB*, *YAP*) but have not been discussed in the text since their diagnostic, prognostic and theranostic relevance are already well described in the WHO CNS5.

First, as illustrated by the cases that we reported and the literature review, there is no morphological and IHC diagnostic specificity for RTK-fused pediatric CNS tumors.

The two *NTRK*-fused CNS tumors (*TPM4-NTRK2* and *ETV6-NTRK3*) that we reported showed very different features characterized by a polymorphous and spindle cell morphology, respectively. The IHC profiles were quite heterogeneous with both neuronal and glial immunophenotypes. These two cases resulted in very different final integrated diagnoses, leading to the conclusion that the presence of an *NTRK* oncogenic fusion alone cannot be used as a diagnostic criterion. The literature review, summarized in Table 1, confirms this hypothesis showing different entities carrying *NTRK* fusions, such as diffuse LGG, MAPK pathway-altered, IHG, PXA and pilocytic astrocytoma [2,14,28,29]. Among the IHGs, the *NTRK*-fused cases have a 5-year OS of 42.9%, which is an intermediate prognosis between the *ALK*-fused and the *ROS1*-fused IHGs [14].

Regarding the *ROS1* fusions, our case with *GOPC-ROS1* fusion exhibited an LG morphology, most closely resembling pilocytic astrocytoma. This diagnostic hypothesis was confirmed by DNA methylation profiling, providing a match with pilocytic astrocytoma MC, albeit with a low score (cs 0.5). The literature review confirms, as previously published by Sievers et al., that these oncogenic fusions have limited diagnostic value because they are not specific to a specific tumor type, though they are most commonly encountered in IHGs [25]. Sievers et al. [25] reported pilocytic astrocytomas and IDH-wt glioblastoma which harbored *ROS1* oncogenic fusion, consistent with our case that has been classified morphologically and epigenetically as a pilocytic astrocytoma.

In the same way, *ALK* and *MET* oncogenic fusions are not pathognomonic to a specific tumor type (Table 1).

Regarding the prognostic implications of these fusions in IHGs, *ALK*, *ROS1* and *NTRK* fusions show slightly different 5-year overall survival (OS) of 53.8%, 25.0% and 42.9%, respectively [14].

Secondly, regarding the oncogenic fusions associated with activation of the MAPK pathway, such as *FGFR3* oncogenic fusions, the case we reported (*FGFR3-TACC3*) was consistent with similar lesions previously described in the literature. Specifically, our case harbored endocrinoïd and “oligodendrocyte-like” morphology, as well as calcifications, which are morphological hallmarks of *FGFR3*-fused gliomas. Although the morphology is reproducible and these fusions have mostly been described in pediatric LGGs [40], the histological grade is not determined by the presence of an FGFR3 oncogenic fusion because these can also be detected in HGGs, albeit mostly in adults [40]. Co-occurrence with other mutations such as *TERT* promoter mutations could have a crucial prognostic impact [40]. As illustrated by our case, which ultimately matched with the diagnosis of DNET (with a cs of 0.7), there is no specific epigenetic MC for *FGFR3*-fused gliomas. This implies that these cases do not represent a specific new histo-molecular entity.

Other rare oncogenic fusions associated with activation of the RAS/MAPK pathway, such as rare *BRAF* and *RAF* oncogenic fusions, have mostly been described in LGGs in the pediatric population (Table 1). The presence of these fusions has poor prognostic value. Therefore, pediatric CNS tumors should be analyzed systematically for fusion genes for optimal management of patients.

Most of the described oncogenic fusions are not specific to a tumor entity: as previously reported, the findings do not support the identification of new histo-molecular entities based on these molecular groups. However, other rare oncogenic fusions such as *MN1*, *PATZ1*, *BCOR* and *CIC* do appear to be diagnostic for specific tumor types. As described above, some are already introduced in the WHO CNS5 such as *MN1* oncogenic fusions defining Astroblastoma *MN1*-altered [14]. Even the *CIC*-rearranged sarcomas have been introduced in the 2021 WHO classification, though our literature review highlighted that it is still not clear if this fusion is specific to sarcomas. This is illustrated by the fact that Sievers et al. described a potential new entity of “High Grade Neuro-Epithelial Tumor (HGNET), *CIC* fusion positive” [67]. In all of these cases, *CIC* fusions were associated with aggressive tumors and early recurrences [67].

Diagnostic challenges remain regarding *PATZ1* and *BCOR* fusions. For *BCOR*-fused CNS tumors, the morphological features seem to be reproducible, but the prognostic impact, in contrary to *BCOR* ITD CNS tumors, remains unknown. Finally, while mostly found in HG tumors, *PATZ1* fusion has also been described in LG tumors, leading to difficulties in providing robust, practical conclusions in routine diagnostics.

From a therapeutic standpoint, the approach differs depending on whether these alterations are present in more or less aggressive tumors. Our cases demonstrate that, despite histology and/or molecular biology suggesting varying degrees of aggressiveness, adjuvant treatment is not always a prerequisite for achieving remission, if complete surgery is achieved. *ALK*-, *ROS1*-, *MET*-, *FGFR*- and *RAF*-fusions provide potential avenues for targeted therapy. However, whereas their inhibitors have been approved or are currently under investigation for the treatment of lung cancer, there is only limited clinical trial data regarding their efficacy in (pediatric) CNS tumors.

In clinical practice, when lacking phase I/II studies with targeted therapies open for these diseases, and the standard of care (maximal safe surgery, chemo-radiotherapy regimens) fails to achieve remission, pediatric oncologists could propose compassionate or extended use programs that are available in different countries [70].

The SACHA French prospective observational study (2020 to 2022) assessed the off-label or compassionate use of targeted drugs and demonstrated that certain targeted drugs, such as *BRAF* or *MEK* inhibitors, are among the most frequently utilized, with pediatric CNS tumors being the main indication [71]. Increasing molecular insights combined with an increased availability of drugs that potentially target these oncogenic fusions will inevitably surge their use.

## 5. Conclusions

In conclusion, the literature review and our reported cohort underline that most of these rare oncogenic fusions are not specific to a single histo-molecular entity among the pediatric CNS tumors. Even within tumors harboring the same oncogenic fusions, a wide range of morphological, molecular and epigenetic entities can be observed. These findings highlight the need for caution when applying the fifth CNS WHO classification, as the vast majority of these fusions are not yet incorporated in the diagnosis, including grade evaluation and DNA methylation classification. Therefore, neuropathologists still have a crucial role to play in integrating these new data. Further studies are needed to improve our understanding and assess diagnostic relevance, as well as determine the clinical implications of these rare oncogenic fusions.

## Figures and Tables

**Table 1:** Rare oncogenic fusions in pediatric CNS tumors.

*Gene Fusion Transcript*	*Pediatric Tumor Type (WHO CNS5 Entity or Potential New Entity)*	*Potential Targeted Therapy*
*ALK*	Diffuse low-grade glioma, MAPK pathway-altered ^*L*^ Infant-type hemispheric glioma ^*E*^	Lorlatinib, Crizotinib, Ceritinib, Alectinib, Brigatinib
*ROS1*	Infant-type hemispheric glioma ^*E*^ Pilocytic astrocytoma ^*L*^ Diffuse pediatric-type high-grade glioma H3-wildtype and IDH-wildtype ^*L*^ Supratentorial ependymoma ^*L*^ Ganglioglioma [25] Dysembryoplastic neuroepithelial tumour [25]	Crizotinib, Entrectinib, Lorlatinib
*NTRK 1-2-3*	Diffuse low-grade glioma, MAPK pathway-altered ^*L*^ Infant-type hemispheric glioma ^*E*^ Pleomorphic xanthoastrocytoma ^*L*^ Pilocytic astrocytoma [2] Ganglioglioma ^*L*^ Supratentorial ependymoma [28,29] Pediatric-type high-grade glioma [28] Desmoplastic infantile ganglioglioma [28,29]	Larotrectinib, Entrectinib, Crizotinib, Cabozantinib
*MET1*	Diffuse low-grade glioma, MAPK pathway-altered ^*L*^ Diffuse pediatric-type high-grade glioma, H3-wildtype and IDH-wildtype ^*L*^ Infant-type hemispheric glioma ^*E*^ Pediatric-type glioneuronal tumors [35]	Capmatinib, Crizotinib, Cabozantinib
*FGFR1*	Extraventricular neurocytoma ^*D*^ Dysembryoplastic neuroepithelial tumor ^*L*^ Ganglioglioma ^*L*^ Diffuse low-grade glioma, MAPK pathway-altered ^*L*^	Regorafenib, Erdafitinib, Rogaratinib, Dovitinib
*FGFR2*	Multinodular and vacuolating neuronal tumor ^*L*^ Ganglioglioma ^*L*^ Polymorphous low-grade neuroepithelial tumor of the young ^*E*^	Infigratinib, Regorafenib, Erdafitinib, Rogaratinib, Dovitinib
*FGFR3*	Diffuse low-grade glioma, MAPK pathway-altered Polymorphous low-grade neuroepithelial tumor of the young ^*E*^ Dysembryoplastic neuroepithelial tumor ^*C*^ Rare in pediatric high-grade glioma [40]	Vofatamab, Regorafenib, Erdafitinib, Rogaratinib, Dovitinib
*BRAF (non-KIAA1549)*	Pleomorphic xanthoastrocytoma [43,45] Pilocytic astrocytoma [43,45] Low-grade glial/glioneuronal tumor NOS [43,45]	Vemurafenib, Dabrafenib, Encorafenib
*RAF1*	Desmoplastic infantile ganglioglioma/desmoplastic infantile astrocytoma ^*L*^ Diffuse leptomeningeal glioneuronal tumor [46] Ganglioglioma ^*L*^ Pleomorphic xanthoastrocytoma ^*L*^ Pilocytic astrocytoma ^*L*^	Selumetinib, Trametinib, Sorafenib
*MN1*	Astroblastoma, MN1-altered ^*E*^ Neuroepithelial tumor, MN1:CXXC5-fused (DKFZ classifier)	-
*PATZ1*	CNS neoplasms with PATZ1 fusions [58] High-grade astrocytoma Ependymoma-, subependymoma-like, low-grade glial/glioneuronal morphology [58,61]	-
*BCOR*	BCOR fused tumors [2,64]	-
*CIC*	High-grade neuroepithelial tumor CIC fusion positive [66] CIC-rearranged sarcoma [14] CNS embryonal tumor [2] Anaplastic pleomorphic xanthoastocytoma [8]	-

*^C^* Case report. *^E^* The fusion is an essential diagnostic criteria according to the WHO CNS5. *^D^* The fusion is a desirable according to the WHO CNS5. *^L^* Described in the literature and cited by the WHO CNS5. WHO: World Health Classification; CNS: Central Nervous System.

## Data Availability

Additional data regarding the cases can be requested from the corresponding author.

## Supplementary Materials

The following supporting information can be downloaded at https://www.mdpi.com/article/10.3390/cancers16193344/s1: File S1: NGS DNA and NGS RNA panels.

## References

[1] Ostrom Q.T., Price M., Neff C., Cioffi G., Waite K.A., Kruchko C., Barnholtz-Sloan J.S. (2022). CBTRUS Statistical Report: Primary Brain and Other Central Nervous System Tumors Diagnosed in the United States in 2015–2019. Neuro Oncol..

[2] Roosen M., Odé Z., Bunt J., Kool M. (2022). The Oncogenic Fusion Landscape in Pediatric CNS Neoplasms. Acta Neuropathol..

[3] Ryall S., Tabori U., Hawkins C. (2020). Pediatric low-grade glioma in the era of molecular diagnostics. Acta Neuropathol. Commun..

[4] Sahm F., Brandner S., Bertero L., Capper D., French P.J., Figarella-Branger D., Giangaspero F., Haberler C., Hegi M.E., Kristensen B.W. (2023). Molecular diagnostic tools for the World Health Organization (WHO) 2021 classification of gliomas, glioneuronal and neuronal tumors; an EANO guideline. Neuro Oncol..

[5] Qaddoumi I., Orisme W., Wen J., Santiago T., Gupta K., Dalton J.D., Tang B., Haupfear K., Punchihewa C., Easton J. (2016). Genetic alterations in uncommon low-grade neuroepithelial tumors: BRAF, FGFR1, and MYB mutations occur at high frequency and align with morphology. Acta Neuropathol..

[6] Andreiuolo F., Varlet P., Tauziède-Espariat A., Jünger S.T., Dörner E., Dreschmann V., Kuchelmeister K., Waha A., Haberler C., Slavc I. (2019). Childhood supratentorial ependymomas with YAP1-MAMLD1 fusion: An entity with characteristic clinical, radiological, cytogenetic and histopathological features. Brain Pathol..

[7] Andreiuolo F., Ferrone C.K., Rajan S., Perry A., Guney E., Cham E., Giannini C., Toland A., Willard N., de Souza A.S. (2024). Molecular and clinicopathologic characteristics of CNS embryonal tumors with BRD4::LEUTX fusion. Acta Neuropathol. Commun..

[8] Lebrun L., Allard-Demoustiez S., Gilis N., Van Campenhout C., Rodesch M., Roman C., Calò P., Lolli V., David P., Fricx C. (2023). Clinicopathological and molecular characterization of a case classified by DNA-methylation profiling as “CNS embryonal tumor with BRD4-LEUTX fusion”. Acta Neuropathol. Commun..

[9] Ahmed M., De Praeter M., Verlooy J., Schoonjans A.S., Dekeyzer S., Vanden Bossche S., Lammens M., Pauwels P. (2022). A case report of a novel NTRK gene fusion in pleomorphic xanthoastrocytoma. Clin. Neuropathol..

[10] Gnekow A.K., Walker D.A., Kandels D., Picton S., Giorgio P., Grill J., Stokland T., Sandstrom P.E., Warmuth-Metz M., Pietsch T. (2017). A European randomised controlled trial of the addition of etoposide to standard vincristine and carboplatin induction as part of an 18-month treatment programme for childhood (≤16 years) low grade glioma—A final report. Eur. J. Cancer.

[11] Clarke M., Mackay A., Ismer B., Pickles J.C., Tatevossian R.G., Newman S., Bale T.A., Stoler I., Izquierdo E., Temelso S. (2020). Infant High-Grade Gliomas Comprise Multiple Subgroups Characterized by Novel Targetable Gene Fusions and Favorable Outcomes. Cancer Discov..

[12] Hallberg B., Palmer R.H. (2016). The role of the ALK receptor in cancer biology. Ann. Oncol..

[13] Tsai C.C., Huang M.H., Fang C.L., Hsieh K.L., Hsieh T.H., Ho W.L., Chang H., Tsai M.L., Kao Y.C., Miser J.S. (2024). An Infant-Type Hemispheric Glioma with SOX5::ALK: A Novel Fusion. J. Natl. Compr. Cancer Netw..

[14] Louis D.N., Perry A., Wesseling P., Brat D.J., Cree I.A., Figarella-Branger D., Hawkins C., Ng H.K., Pfister S.M., Reifenberger G. (2021). The 2021 WHO Classification of Tumors of the Central Nervous System: A summary. Neuro Oncol..

[15] Guerreiro Stucklin A.S., Ryall S., Fukuoka K., Zapotocky M., Lassaletta A., Li C., Bridge T., Kim B., Arnoldo A., Kowalski P.E. (2019). Alterations in ALK/ROS1/NTRK/MET drive a group of infantile hemispheric gliomas. Nat. Commun..

[16] Wrona A. (2019). Management of CNS disease in ALK-positive non-small cell lung cancer: Is whole brain radiotherapy still needed?. Cancer Radiother..

[17] Lai M., Li S., Li H., Hu Q., Li J., Zhou J., Ai R., Zhen J., Zhou Z., Wang L. (2023). Lorlatinib for ALK-fused, infant-type hemispheric glioma with lung metastasis: A case report. Ann. Clin. Transl. Neurol..

[18] Pearce J., Khabra K., Nanji H., Stone J., Powell K., Martin D., Zebian B., Hettige S., Reisz Z., Bodi I. (2021). High grade gliomas in young children: The South Thames Neuro-Oncology unit experience and recent advances in molecular biology and targeted therapies. Pediatr. Hematol. Oncol..

[19] Desai A.V., Robinson G.W., Gauvain K., Basu E.M., Macy M.E., Maese L., Whipple N.S., Sabnis A.J., Foster J.H., Shusterman S. (2022). Entrectinib in children and young adults with solid or primary CNS tumors harboring NTRK, ROS1, or ALK aberrations (STARTRK-NG). Neuro Oncol..

[20] Meredith D.M., Cooley L.D., Dubuc A., Morrissette J., Sussman R.T., Nasrallah M.P., Rathbun P., Yap K.L., Wadhwani N., Bao L. (2023). ROS1 Alterations as a Potential Driver of Gliomas in Infant, Pediatric, and Adult Patients. Mod. Pathol..

[21] Berlanga P., Pierron G., Lacroix L., Chicard M., Adam de Beaumais T., Marchais A., Harttrampf A.C., Iddir Y., Larive A., Soriano Fernandez A. (2022). The European MAPPYACTS Trial: Precision Medicine Program in Pediatric and Adolescent Patients with Recurrent Malignancies. Cancer Discov..

[22] van Tilburg C.M., Pfaff E., Pajtler K.W., Langenberg K.P.S., Fiesel P., Jones B.C., Balasubramanian G.P., Stark S., Johann P.D., Blattner-Johnson M. (2021). The Pediatric Precision Oncology INFORM Registry: Clinical Outcome and Benefit for Patients with Very High-Evidence Targets. Cancer Discov..

[23] Richardson T.E., Tang K., Vasudevaraja V., Serrano J., William C.M., Mirchia K., Pierson C.R., Leonard J.R., AbdelBaki M.S., Schieffer K.M. (2019). GOPC-ROS1 Fusion Due to Microdeletion at 6q22 Is an Oncogenic Driver in a Subset of Pediatric Gliomas and Glioneuronal Tumors. J. Neuropathol. Exp. Neurol..

[24] Deland L., Keane S., Bontell T.O., Fagman H., Sjögren H., Lind A.E., Carén H., Tisell M., Nilsson J.A., Ejeskär K. (2022). Novel TPR::ROS1 Fusion Gene Activates MAPK, PI3K and JAK/STAT Signaling in an Infant-type Pediatric Glioma. Cancer Genom. Proteom..

[25] Sievers P., Stichel D., Sill M., Schrimpf D., Sturm D., Selt F., Ecker J., Kazdal D., Miele E., Kranendonk M.E.G. (2021). GOPC:ROS1 and other ROS1 fusions represent a rare but recurrent drug target in a variety of glioma types. Acta Neuropathol..

[26] Papusha L., Zaytseva M., Panferova A., Druy A., Valiakhmetova A., Artemov A., Salnikova E., Kislyakov A., Imyanitov E., Karachunsky A. (2022). Two clinically distinct cases of infant hemispheric glioma carrying ZCCHC8:ROS1 fusion and responding to entrectinib. Neuro Oncol..

[27] Lang S.S., Kumar N.K., Madsen P., Gajjar A.A., Gajjar E., Resnick A.C., Storm P.B. (2022). Neurotrophic tyrosine receptor kinase fusion in pediatric central nervous system tumors. Cancer Genet..

[28] Torre M., Vasudevaraja V., Serrano J., DeLorenzo M., Malinowski S., Blandin A.F., Pages M., Ligon A.H., Dong F., Meredith D.M. (2020). Molecular and clinicopathologic features of gliomas harboring NTRK fusions. Acta Neuropathol. Commun..

[29] Gambella A., Senetta R., Collemi G., Vallero S.G., Monticelli M., Cofano F., Zeppa P., Garbossa D., Pellerino A., Rudà R. (2020). NTRK Fusions in Central Nervous System Tumors: A Rare, but Worthy Target. Int. J. Mol. Sci..

[30] Doz F., van Tilburg C.M., Geoerger B., Højgaard M., Øra I., Boni V., Capra M., Chisholm J., Chung H.C., DuBois S.G. (2022). Efficacy and safety of larotrectinib in TRK fusion-positive primary central nervous system tumors. Neuro Oncol..

[31] Papusha L., Zaytseva M., Druy A., Valiakhmetova A., Yasko L., Salnikova E., Shekhtman A., Karachunsky A., Maschan A., Hwang E.I. (2021). The experience of successful treatment of ETV6-NTRK3-positive infant glioblastoma with entrectinib. Neuro-Oncol. Adv..

[32] Riedmeier M., Stock A., Krauß J., Sahm F., Jones D.T.W., Sturm D., Kramm C.M., Eyrich M., Härtel C., Schlegel S. (2021). Spontaneous regression of a congenital high-grade glioma—A case report. Neuro-Oncol. Adv..

[33] International Cancer Genome Consortium PedBrain Tumor Project (2016). Recurrent MET fusion genes represent a drug target in pediatric glioblastoma. Nat. Med..

[34] Chapman N., Greenwald J., Suddock J., Xu D., Markowitz A., Humphrey M., Cotter J.A., Krieger M.D., Hawes D., Ji J. (2024). Clinical, pathologic, and genomic characteristics of two pediatric glioneuronal tumors with a CLIP2::MET fusion. Acta Neuropathol. Commun..

[35] Sievers P., Sill M., Schrimpf D., Friedel D., Sturm D., Gardberg M., Kurian K.M., Krskova L., Vicha A., Schaller T. (2022). Epigenetic profiling reveals a subset of pediatric-type glioneuronal tumors characterized by oncogenic gene fusions involving several targetable kinases. Acta Neuropathol..

[36] Zuckermann M., He C., Andrews J., Bagchi A., Sloan-Henry R., Bianski B., Xie J., Wang Y., Twarog N., Onar-Thomas A. (2024). Capmatinib is an effective treatment for MET-fusion driven pediatric high-grade glioma and synergizes with radiotherapy. Mol. Cancer.

[37] Bale T.A. (2020). FGFR-gene family alterations in low-grade neuroepithelial tumors. Acta Neuropathol. Commun..

[38] Di Stefano A.L., Picca A., Saragoussi E., Bielle F., Ducray F., Villa C., Eoli M., Paterra R., Bellu L., Mathon B. (2020). Clinical, molecular, and radiomic profile of gliomas with FGFR3-TACC3 fusions. Neuro Oncol..

[39] Mata D.A., Benhamida J.K., Lin A.L., Vanderbilt C.M., Yang S.R., Villafania L.B., Ferguson D.C., Jonsson P., Miller A.M., Tabar V. (2020). Genetic and epigenetic landscape of IDH-wildtype glioblastomas with FGFR3-TACC3 fusions. Acta Neuropathol. Commun..

[40] Métais A., Tauziède-Espariat A., Garcia J., Appay R., Uro-Coste E., Meyronet D., Maurage C.A., Vandenbos F., Rigau V., Chiforeanu D.C. (2023). Clinico-pathological and epigenetic heterogeneity of diffuse gliomas with FGFR3::TACC3 fusion. Acta Neuropathol. Commun..

[41] Sievers P., Stichel D., Schrimpf D., Sahm F., Koelsche C., Reuss D.E., Wefers A.K., Reinhardt A., Huang K., Ebrahimi A. (2018). FGFR1:TACC1 fusion is a frequent event in molecularly defined extraventricular neurocytoma. Acta Neuropathol..

[42] Mongiardi M.P., Pallini R., D’Alessandris Q.G., Levi A., Falchetti M.L. (2024). Regorafenib and glioblastoma: A literature review of preclinical studies, molecular mechanisms and clinical effectiveness. Expert Rev. Mol. Med..

[43] Zhang J., Wu G., Miller C.P., Tatevossian R.G., Dalton J.D., Tang B., Orisme W., Punchihewa C., Parker M., Qaddoumi I. (2013). Whole-genome sequencing identifies genetic alterations in pediatric low-grade gliomas. Nat. Genet..

[44] Lind K.T., Chatwin H.V., DeSisto J., Coleman P., Sanford B., Donson A.M., Davies K.D., Willard N., Ewing C.A., Knox A.J. (2021). Novel RAF Fusions in Pediatric Low-Grade Gliomas Demonstrate MAPK Pathway Activation. J. Neuropathol. Exp. Neurol..

[45] Daoud E.V., Wachsmann M., Richardson T.E., Mella D., Pan E., Schwarzbach A., Oliver D., Hatanpaa K.J. (2019). Spinal Pleomorphic Xanthoastrocytoma With a QKI-RAF1 Fusion. J. Neuropathol. Exp. Neurol..

[46] Benhamida J.K., Harmsen H.J., Ma D., William C.M., Li B.K., Villafania L., Sukhadia P., Mullaney K.A., Dewan M.C., Vakiani E. (2023). Recurrent TRAK1::RAF1 Fusions in pediatric low-grade gliomas. Brain Pathol..

[47] Yde C.W., Sehested A., Mateu-Regué À., Østrup O., Scheie D., Nysom K., Nielsen F.C., Rossing M. (2016). A new NFIA:RAF1 fusion activating the MAPK pathway in pilocytic astrocytoma. Cancer Genet..

[48] Riedel S.S., Lu C., Xie H.M., Nestler K., Vermunt M.W., Lenard A., Bennett L., Speck N.A., Hanamura I., Lessard J.A. (2021). Intrinsically disordered Meningioma-1 stabilizes the BAF complex to cause AML. Mol. Cell.

[49] Heuser M., Argiropoulos B., Kuchenbauer F., Yung E., Piper J., Fung S., Schlenk R.F., Dohner K., Hinrichsen T., Rudolph C. (2007). MN1 overexpression induces acute myeloid leukemia in mice and predicts ATRA resistance in patients with AML. Blood.

[50] Libbrecht C., Xie H.M., Kingsley M.C., Haladyna J.N., Riedel S.S., Alikarami F., Lenard A., McGeehan G.M., Ernst P., Bernt K.M. (2021). Menin is necessary for long term maintenance of meningioma-1 driven leukemia. Leukemia.

[51] Palma Milla C., Patricia P.M., Lezana J.M., Cruz J., Quesada J.F., Vila S., Álvarez-Mora I., Arteche-López A., Gómez-Manjón I., Sánchez M.T. (2023). A Novel Pathogenic Variant in the MN1 Gene in a Patient Presenting with Rhombencephalosynapsis and Craniofacial Anomalies, Expanding MN1 C-terminal Truncation Syndrome. J. Pediatr. Genet..

[52] Kim H., Lee K., Phi J.H., Paek S.H., Yun H., Choi S.H., Park S.H. (2023). Neuroepithelial tumor with EWSR1::PATZ1 fusion: A literature review. J. Neuropathol. Exp. Neurol..

[53] Schmitt-Hoffner F., Gojo J., Mauermann M., vonHoff K., Sill M., Korshunov A., Stichel D., Sahm F., Jäger N., Pfister S.M. (2022). Molecular and clinical characterization of the new WHO entity ‘Astroblastoma, MN1 altered’ and its molecular subgroups. Cancer Res..

[54] Lehman N.L., Usubalieva A., Lin T., Allen S.J., Tran Q.T., Mobley B.C., McLendon R.E., Schniederjan M.J., Georgescu M.M., Couce M. (2019). Genomic analysis demonstrates that histologically-defined astroblastomas are molecularly heterogeneous and that tumors with MN1 rearrangement exhibit the most favorable prognosis. Acta Neuropathol. Commun..

[55] Lubieniecki F., Vazquez V., Lamas G.S., Camarero S., Nuñez F.J., Baroni L., Schüller U., Alderete D. (2023). The spectrum of morphological findings in pediatric central nervous system MN1-fusion-positive neuroepithelial tumors. Childs Nerv. Syst..

[56] Wood M.D., Tihan T., Perry A., Chacko G., Turner C., Pu C., Payne C., Yu A., Bannykh S.I., Solomon D.A. (2018). Multimodal molecular analysis of astroblastoma enables reclassification of most cases into more specific molecular entities. Brain Pathol..

[57] Frederico S.C., Vera E., Abdullaev Z., Acquaye A., Aldape K., Boris L., Briceno N., Choi A., Christ A., Cooper D. (2023). Heterogeneous clinicopathological findings and patient-reported outcomes in adults with MN1-altered CNS tumors: A case report and systematic literature review. Front. Oncol..

[58] Alhalabi K.T., Stichel D., Sievers P., Peterziel H., Sommerkamp A.C., Sturm D., Wittmann A., Sill M., Jäger N., Beck P. (2021). PATZ1 fusions define a novel molecularly distinct neuroepithelial tumor entity with a broad histological spectrum. Acta Neuropathol..

[59] Siegfried A., Rousseau A., Maurage C.A., Pericart S., Nicaise Y., Escudie F., Grand D., Delrieu A., Gomez-Brouchet A., Le Guellec S. (2019). EWSR1-PATZ1 gene fusion may define a new glioneuronal tumor entity. Brain Pathol..

[60] Costoya J.A. (2007). Functional analysis of the role of POK transcriptional repressors. Brief. Funct. Genom. Proteom..

[61] Rossi S., Barresi S., Colafati G.S., Genovese S., Tancredi C., Costabile V., Patrizi S., Giovannoni I., Asioli S., Poliani P.L. (2024). PATZ1-Rearranged Tumors of the Central Nervous System: Characterization of a Pediatric Series of Seven Cases. Mod. Pathol..

[62] Wamstad J.A., Bardwell V.J. (2007). Characterization of Bcor expression in mouse development. Gene Expr. Patterns.

[63] Huynh K.D., Fischle W., Verdin E., Bardwell V.J. (2000). BCoR, a novel corepressor involved in BCL-6 repression. Genes Dev..

[64] Tauziède-Espariat A., Uro-Coste E., Sievers P., Nicaise Y., Mariet C., Siegfried A., Pierron G., Guillemot D., Benzakoun J., Pallud J. (2023). CNS tumor with EP300::BCOR fusion: Discussing its prevalence in adult population. Acta Neuropathol. Commun..

[65] Ebrahimi A., Waha A., Schittenhelm J., Gohla G., Schuhmann M.U., Pietsch T. (2024). BCOR::CREBBP fusion in malignant neuroepithelial tumor of CNS expands the spectrum of methylation class CNS tumor with BCOR/BCOR(L1)-fusion. Acta Neuropathol. Commun..

[66] Sturm D., Orr B.A., Toprak U.H., Hovestadt V., Jones D.T.W., Capper D., Sill M., Buchhalter I., Northcott P.A., Leis I. (2016). New Brain Tumor Entities Emerge from Molecular Classification of CNS-PNETs. Cell.

[67] Sievers P., Sill M., Schrimpf D., Abdullaev Z., Donson A.M., Lake J.A., Friedel D., Scheie D., Tynninen O., Rauramaa T. (2023). Pediatric-type high-grade neuroepithelial tumors with CIC gene fusion share a common DNA methylation signature. NPJ Precis. Oncol..

[68] Lake J.A., Donson A.M., Prince E., Davies K.D., Nellan A., Green A.L., Mulcahy Levy J., Dorris K., Vibhakar R., Hankinson T.C. (2020). Targeted fusion analysis can aid in the classification and treatment of pediatric glioma, ependymoma, and glioneuronal tumors. Pediatr. Blood Cancer.

[69] Chiang J., Bagchi A., Li X., Dhanda S.K., Huang J., Pinto S.N., Sioson E., Dalton J., Tatevossian R.G., Jia S. (2024). High-grade glioma in infants and young children is histologically, molecularly, and clinically diverse: Results from the SJYC07 trial and institutional experience. Neuro Oncol..

[70] Perwein T., Giese B., Nussbaumer G., von Bueren A.O., van Buiren M., Benesch M., Kramm C.M. (2023). How I treat recurrent pediatric high-grade glioma (pHGG): A Europe-wide survey study. J. Neuro-Oncol..

[71] Berlanga P., Ndounga-Diakou L.A., Corradini N., Ducassou S., Strullu M., De Carli E., Andre N., Entz-Werle N., Defachelles A.S., Roumy M. (2022). Securing access to innovative anticancer therapies for children, adolescents, and young adults outside clinical trials: The SACHA study of the French Society of Pediatric Oncology (SFCE). J. Clin. Oncol..

