# Supplementary material for: Rare Oncogenic Fusions in Pediatric Central Nervous System Tumors: A Case Series and Literature Review
Source: Cancers (Basel). 2024 Sep 30;16(19):3344. doi: 10.3390/cancers16193344 (PMC11475864; doi:10.3390/cancers16193344)
Supplement: Supplementary file 1 [file cancers-16-03344-s001.zip › cancers-3220066-supplementary.pdf]

S1: NGS DNA and NGS RNA panels: next-generation sequencing (NGS) DNA panel includes gene/exon alterations: ACVR1, ATRX, BRAF, CDK6, CDKN2A, CIC, DAXX, EGFR, FUBP1, H3F3A, H3F3B, HIST1H3B, HIST1H3C, IDH1, IDH2, MET, NF1, PIK3CA, PDGFRA, PTEN, TERTp, TP53. Copy number variation analysis was restricted to the analysis of CDKN2a deletion and EGFR amplification. NGS RNA panel includes gene fusion transcripts: ABL1, AKT3, ALK, AXL, BRAF, ERBB2, EGFR, ERG, ETV1-4-5, FGFR1-2-3, MET, NTRK1-2-3, PDGFRA, PPARG, RAF1, RET, ROS.
